# P-1673. Ferritin, Procalcitonin and Lipid Profile as Biomarkers in Tick Borne Diseases: A 10-year Retrospective Study

**DOI:** 10.1093/ofid/ofaf695.1847

**Published:** 2026-01-11

**Authors:** Abdullah Khan Zada, Rudline G Zamor, Luis A Marcos

**Affiliations:** Stony Brook University Hospital, Stony Brook, NY; Stony Brook University Hospital, Stony Brook, NY; Renaissance School of Medicine at Stony Brook University, Stony Brook, New York

## Abstract

**Background:**

Tick borne diseases (TBD) represent a significant public health concern with increasing incidence across the US. This study aims to evaluate and compare laboratory markers with particular emphasis on ferritin levels, procalcitonin and lipid profile across TBD to enhance diagnostic accuracy and inform therapeutic decision making.

**Table 1:**
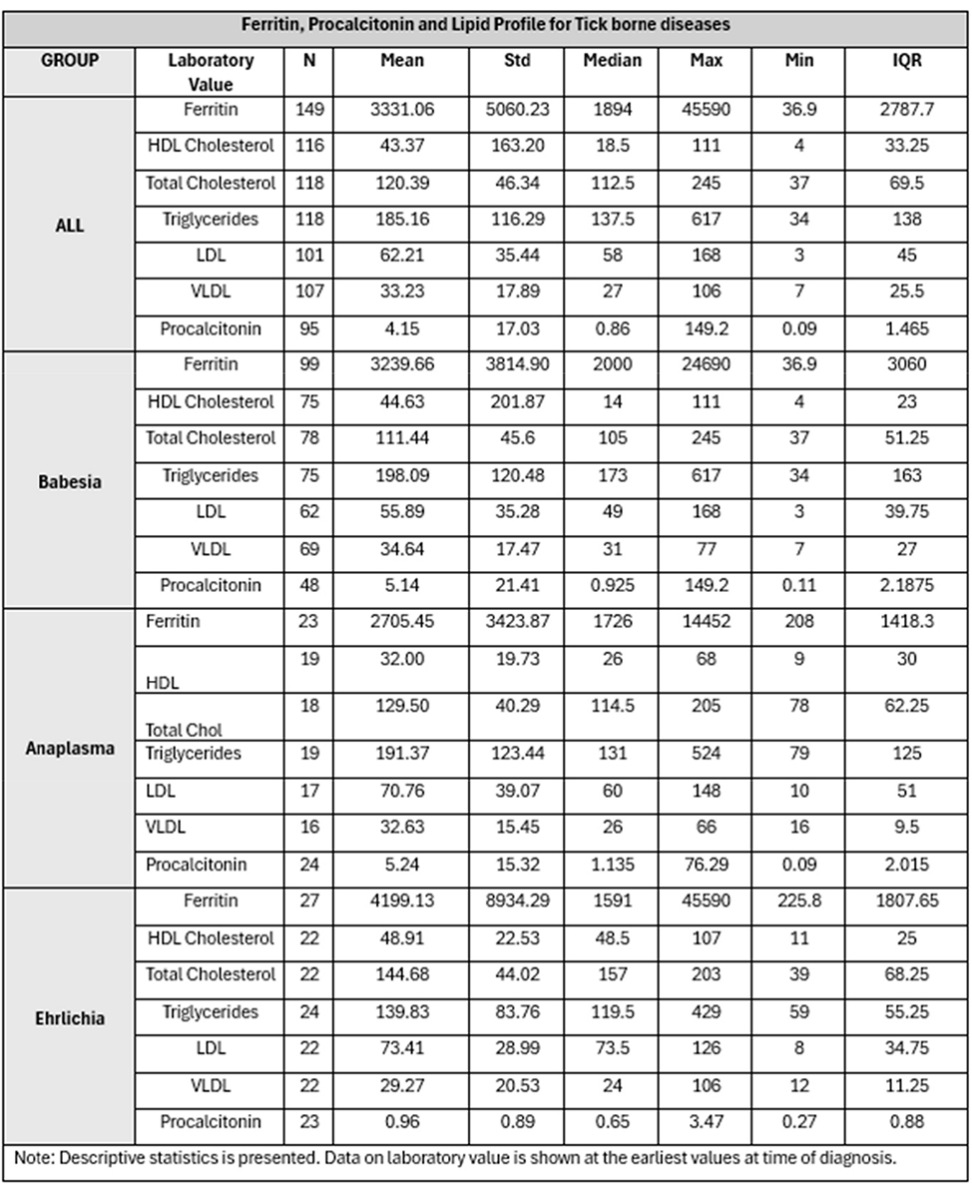
Descriptive table of mean, median values of ferritin, procalcitonin and lipid profile in tick borne diseases

**Figure 2:**
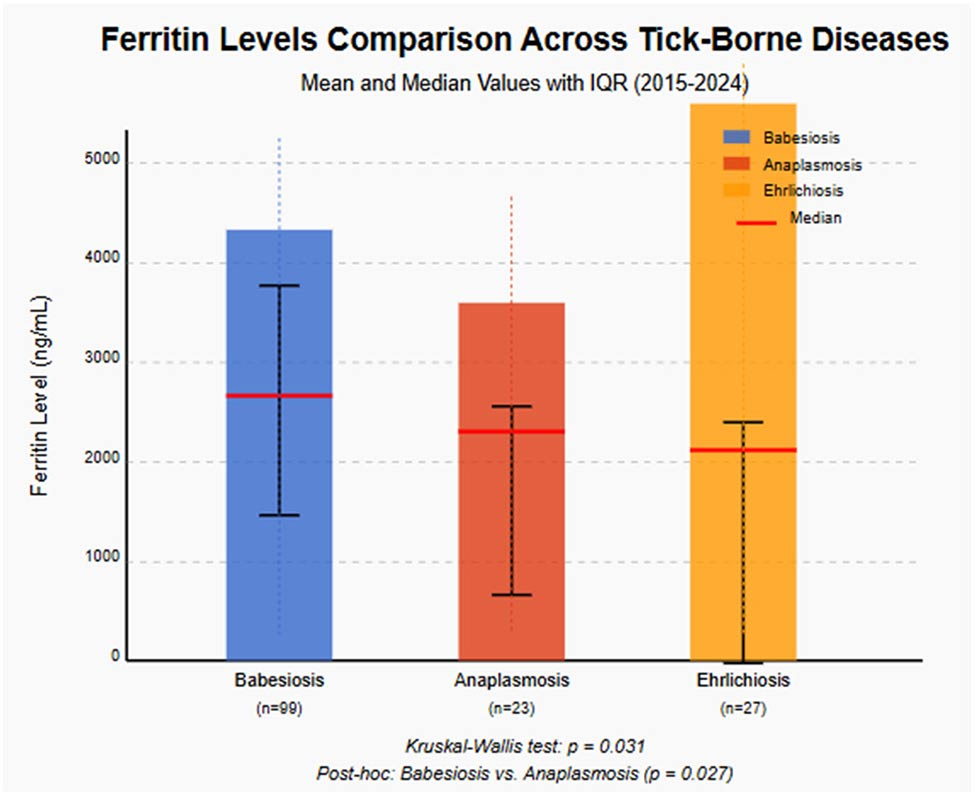
Comparison of ferritin across tick borne diseases

**Methods:**

We conducted a retrospective analysis of 429 patients (274 babesiosis, 69 anaplasmosis, 86 ehrlichiosis) diagnosed by PCR at Stony Brook University Hospital between 2015 and 2024. Inclusion criteria for the analysis were those patients who had the following biomarkers during admission: ferritin, procalcitonin and lipid profiles. Statistical analysis applied to categorical variables was by X2 and continuous variables was by Student’s t test. A p-value of less than 0.05 was considered statistically significant.

**Figure 3:**
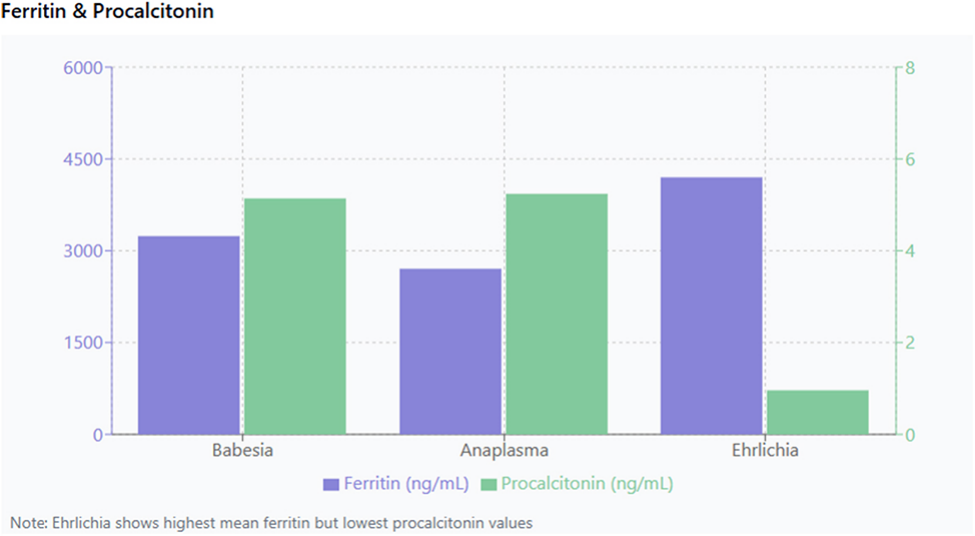
Comparison of ferritin and procalcitonin across tick borne diseases

**Figure 4:**
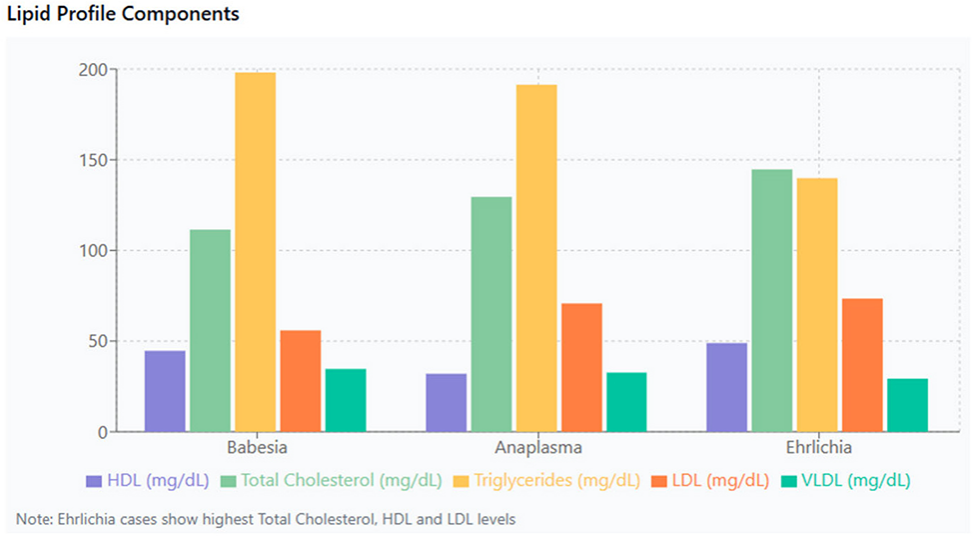
Comparison of lipid profile across tick borne diseases

**Results:**

Hyperferritinemia was a consistent feature across all tick borne diseases. Mean ferritin values were 3239.66 ng/mL for babesiosis, 2705.45 ng/mL for anaplasmosis and 4199.13 ng/mL for ehrlichiosis. Procalcitonin was markedly elevated in babesiosis (mean 5.14 ng/mL) and anaplasmosis (mean 5.24 ng/mL) compared to ehrlichiosis (mean 0.96 ng/mL) (p=0.018). Total cholesterol was lowest in babesiosis (mean 111.44 mg/dL) compared to anaplasmosis (mean 129.50 mg/dL) and ehrlichiosis (mean 144.68 mg/dL) (p< 0.001). HDL cholesterol levels were depressed across all groups, but most pronounced in anaplasmosis (mean 32.00 mg/dL) compared to babesiosis (mean 44.63 mg/dL) and ehrlichiosis (mean 48.91 mg/dL) (p=0.009). Triglyceride levels were elevated, highest in babesiosis (mean 198.09 mg/dL), anaplasmosis (191.37 mg/dL) and ehrlichiosis (139.83 mg/dL) (p=0.029). LDL cholesterol were low, with mean values of 55.89 mg/dL, 70.76 mg/dL, and 73.41 mg/dL for babesiosis, anaplasmosis, and ehrlichiosis, respectively (p=0.011).

**Conclusion:**

This study demonstrates that hyperferritinemia, procalcitonin elevation and dyslipidemia provide distinct laboratory signatures for TBD. Ferritin should be routinely measured in suspected tick borne disease cases as extreme elevation may support diagnosis and potentially indicate more severe disease.

**Disclosures:**

All Authors: No reported disclosures

